# COVID-19 Coinfection With Mucormycosis in a Diabetic Patient

**DOI:** 10.7759/cureus.15820

**Published:** 2021-06-22

**Authors:** Roopa R, Malarkodi Thanthoni, Aravind S Warrier

**Affiliations:** 1 Oral Medicine and Radiology, Sri Ramachandra Faculty of Dental Sciences, Sri Ramachandra Institute of Higher Education and Research, Chennai, IND

**Keywords:** covid-19, coronavirus, mucormycosis, diabetes mellitus, opportunistic fungal infection

## Abstract

Severe acute respiratory syndrome coronavirus 2 (SARS-CoV-2), a form of β-Coronavirus of the Coronaviridae family, has been causing infection among humans worldwide leading to a pandemic emergency. New strains of SARS-CoV-2 have been evolving unceasingly, presenting with various systemic and oral manifestations. There has been an increase in the incidence of secondary infection in the coronavirus infected individual either due to pre-existing factors or the virus by itself is causing such infection, which is still unclear. As it is already known, immunocompromised and uncontrolled diabetic patients have an increased chance of developing mucormycosis. Herein, we report a case who presented with a swelling in the left cheek, eye, and avascular necrosis intraorally, post COVID-19 infection.

## Introduction

On February 11, 2020, the World Health Organization (WHO) named severe acute respiratory syndrome coronavirus 2 (SARS-CoV-2) causing pneumonia as coronavirus disease 2019 (COVID-19). SARS-CoV-2 infection is different morphologically from the previously known coronavirus which caused pneumonia, such as SARS-CoV and Middle East respiratory syndrome coronavirus (MERS-CoV). On March 11, 2020, COVID-19 was declared a pandemic by WHO [1,2]. As in April 2021, COVID-19 infection was confirmed in 135 million people with a fatality of 2.92 million across the world. The morbidity and mortality of COVID-19 disease are increasing with its infection with other microorganisms, such as viruses, bacteria, and fungi. The lungs are the primary site of infection for COVID-19, with symptoms ranging from flu-like symptoms to fulminant pneumonia and severe respiratory distress [3]. Several oral manifestations have been described till now. Mucormycosis is one such secondary infection reported in few patients [4]. Mucormycosis is a fungal infection previously known as zygomycosis and is caused by mucormycetes, which affect the sinus, lungs, or skin through inhalation of spores from the air or via penetration of skin through a cut or burn. Mucormycosis is a rare but devastating disease and more commonly seen in uncontrolled diabetic patients [5]. In this article, we are reporting a case of mucormycosis arising as a post-COVID complication.

## Case presentation

**Figure 1:**
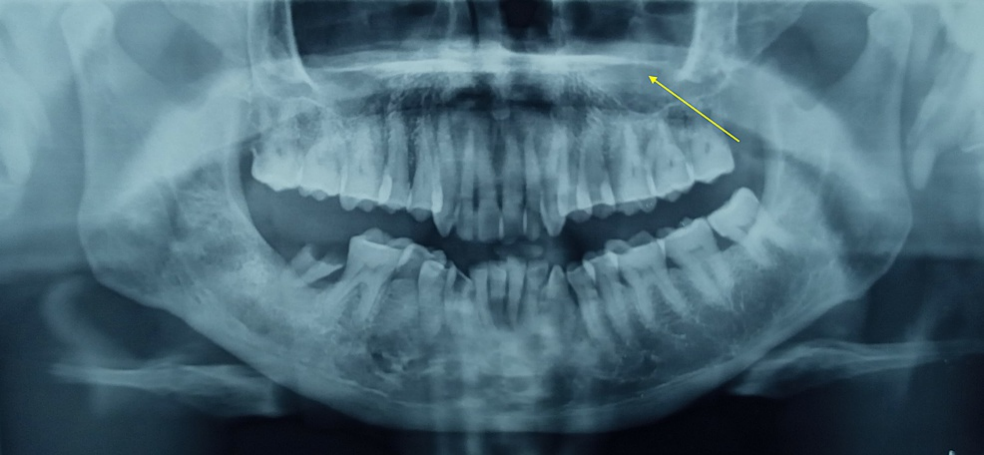
OPT reveals opacification in the left maxillary sinus (yellow arrow). OPT - Orthopantomogram

**Figure 2:**
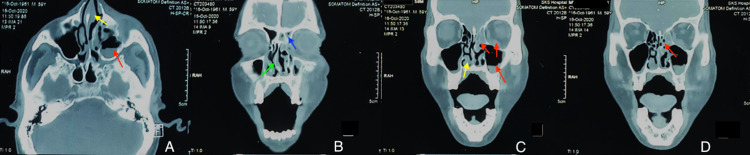
MDCT axial section (A) and coronal section (B-D) of facial bones. Nasal mucosal thickening (yellow arrow), mucosal thickening in left frontal sinus (blue arrow), mucosal thickening and fluid in the left maxillary, ethmoidal sinus and inflamed inferior rectus (red arrow), and deviation of nasal septum ( green arrow). MDCT: Multidetector computed tomography

**Figure 3:**
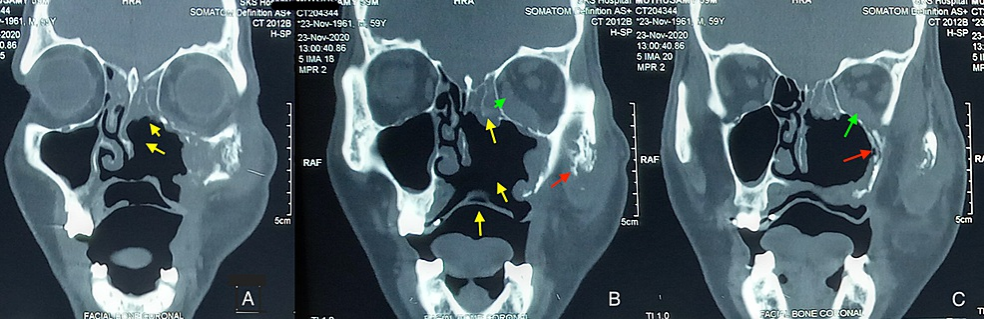
Post sequestrectomy MDCT coronal section of facial bones (A-C) reveals changes showing osseous defects in the hard palate, left ethmoidal sinus, medial wall and floor of left maxillary sinus (yellow arrow), ill-defined bony erosion involving the lateral wall of maxillary sinus, left zygomatico-maxillary process (red arrow), and inflammation of inferior and medial rectus (green arrow). MDCT: multidetector CT scan

A 59-year-old male presented with a complaint of pain in the left cheek, eye, and head for a duration of one month. Before one month, the patient had developed fever for four days due to which he was subjected to a real-time reverse transcription-polymerase chain reaction (RT-PCR) for COVID-19 and was found to be positive. He was hospitalized for the same for 20 days and was under O_^2^_ prone ventilation, IV fluids, injections, remedisvir, piperacillin + tazobac, pantoprazole 40mg, acetylcysteine, enoxaparin 40mg/0.4mL and Vitamin C along with tablets aspirin 75mg, pirfenidone 200mg, atorvastatin 40mg, multivitamin, Vitamin D3 2000, ivermectin 12mg, glipizide 10mg, and supportive therapy. During treatment, the patient complained of mobility and pain in the teeth with pus discharge from gums but the patient was advised to undergo dental management after getting treated for COVID-19. The patient was quarantined for two weeks post-discharge after which he had visited a private dental clinic where he was advised for orthopantomogram (OPT) and computed tomography (CT) imaging (Figures 1 and 2A-2D). OPT revealed opacification in the left maxillary sinus. Multidetector CT (MDCT) scan of facial bones revealed bilateral maxillary, left ethmoidal and frontal sinusitis. Ill-defined bony erosion was seen involving the walls of the left maxillary antrum, hard palate and the floor of the right maxillary antrum, features were highly suggestive of osteomyelitis. The patient was prescribed tablet clindamycin 300mg, piroxicam 20mg and three days later, maxillary sequestrectomy was done under local anesthesia in the same private clinic. Histopathological analysis confirmed it to be fungal osteomyelitis following which the patient was prescribed tablet ketoconazole 400mg once daily. The next day patient has developed intermittent sharp pain, radiating to the left side of the head aggravating the left side on lying. The same day, the patient has developed a swelling on the left side of the face for which the patient was advised to continue the same medication for three more days. Subsequently, the patient was advised to get MDCT as the symptoms did not subside for four weeks while the patient's medical history reveals type II Diabetes mellitus for the past six years and is under medication. The patient was smoking cigarettes for two years and claimed to have quit the habit eight years back. Post sequestrectomy MDCT (Figures 3A-3C) revealed postoperative changes with osseous defects in the remnants of left maxilla, zygomatico-maxillary process, pterygoid process, nasal bone and anteriorly in the midline of the ethmoidal bone and his HbA1c level was 11.6%. The patient reported to our department with the above complaints and reports.

**Figure 4:**
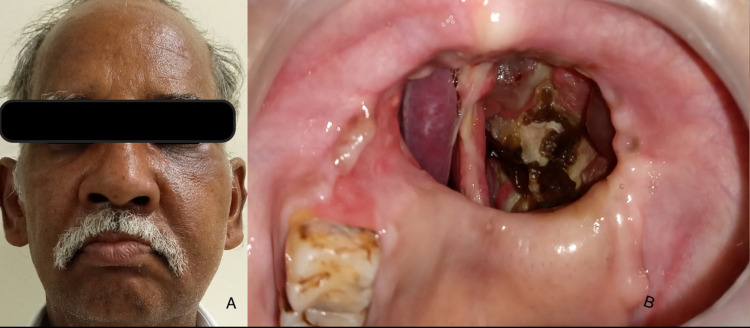
(A) Diffuse swelling involving the left lower eyelid and left middle third of face. (B) Surgical fistula involving the left maxilla with necrotic bone.

Extraoral examination revealed a single diffuse swelling on the left side of the face in the zygoma and eye region of size approximately 3×4 cm in diameter involving the lower eyelid, malar region, lateral wall of the nose (Figures 4A-4D). The surface over the swelling appeared normal with no secondary changes. It was soft, compressible, and severely tender on palpation. The patient had pain with the movement of the left eyeball. No evidence of paraesthesia. Intraoral examination revealed evidence of obturator in the left maxilla with missing left maxilla extending from 15 to 28. The surgical fistula was evident in the left maxilla with necrotic bone on the lateral, medial, and superior wall of the maxillary sinus.

Based on the clinical presentation, history, previous biopsy report, and CT imaging, the diagnosis of residual rhino-orbital mucormycosis of the left maxillary sinus was given. The patient was admitted and started on tablet amphotericin B with a gradual increase in the dosage up to 200mg for 15 days, simultaneously diabetologist opinion and ophthalmologist opinion was sought to control his blood glucose levels and to see the extent of eye involvement. After 15 days, the patient was given Tablet Posaconazole 300 mg once daily. The patient is under regular follow-up and shows signs of healing.

## Discussion

In the present condition, a novel β-coronavirus COVID-19 has been affecting individuals globally at various degrees. Coronavirus is a pleomorphic, single-stranded RNA (ssRNA) and is a part of the Coronaviridae family. Its Coronavirinae subfamily has four genera, i.e., α-CoV, β-CoV, γ-CoV, and δ-CoV (alpha-coronavirus, beta-coronavirus, gamma-coronavirus, and delta-coronavirus). The α-CoV and β-CoV are known to infect humans, while δ-CoV and γ-CoV genera are known to infect pigs and avians. Historically, in 2002, SARS-CoV infection began causing SARS, and in 2012, MERS-CoV infection began causing MERS. SARS-CoV, MERS-CoV, and SARS-CoV-2 fall under beta-coronavirus genera. COVID-19, which is caused by SARS-CoV-2 has been shown to have similar sequencing of 79% with SARS-CoV and 50% with MERS-CoV identity. The difference in these viruses is caused by the difference in gene order, gene expression, nucleotide number, and its sequence [2,6]. Few mutations have been occurring in the SARS-CoV-2 and on analyzing across the globe, out of 220 sequences, eight mutations were repetitive. One such mutation was in the RNA-dependent RNA polymerase gene. These mutations suggest that there is a continuous evolution of the viral strains worldwide [2].

The pathogenesis is unclear in the case of SARS-CoV-2 infection in humans. SARS-CoV-2 is transmitted through aerosols and droplets and reaches the lungs through the naso-oral cavity. When inhaled, the S protein of the SARS-CoV-2 binds with the ACE2 receptor, causing the virus entry into the host cell. The ACE2 receptor is evident on pulmonary epithelial cells and is considered a functional receptor of SARS-CoV-2. Once the virus enters the cell, a positive single stranded RNA is released, which undergoes transcription and forms negative-stranded RNA. This newly formed RNA undergoes translation and produces two polyproteins, which create the viral RNA polymerase. The newly formed RNA combines with capsid protein and forms the nucleocapsid and gets bounded in the endoplasmic reticulum Golgi intermediate compartment (ERGIC). The vesicles loaded with the viral particle are fused to the cell membrane and transferred to extracellular space through exocytosis for viral shedding. These new viral particles invade the adjacent epithelial and can be released in the respiratory droplets into the surrounding environment [2,6].

In the phase when the virus enters the nasal epithelium through aerosols or droplets, it undergoes replication. Though the individual is asymptomatic with a low viral load, they are highly infectious. This phase lasts for few days and can be detected in nasal swab testing. When the virus enters the upper respiratory tract, symptoms like dry cough, fever, and malaise are manifested. There is a great immune response in this phase and for many individuals, the infection does not progress beyond this phase. When the virus enters the lower respiratory tract, it replicates and produces viral nucleocapsids following which inflammatory markers and cytokines are released. This cytokine storm fights off the virus and in turn causes inflammation to the lungs leading to lung injury. The virus-infected cells undergo apoptosis during which new viral particles are released into the surrounding tissue affecting the adjacent epithelial cells. The cyclical process of viral release and inflammation causes diffuse alveolar damage leading to acute respiratory distress syndrome [6].

The incubation period of COVID-19 infection is 2-14 days [2] commonly affecting the 40-70 years old age group. During this period, the individual can be highly contagious. They report symptoms such as breathlessness, fever, dry cough, body pain, loss of taste, smell, malaise, vomiting, loose stools, abdominal pain, and some are asymptomatic with mild to moderate disease [6,7]. In the present pandemic situation, studies from Italy and China revealed severe COVID-19 infection with increased mortality rates in older patients with a chronic disease like diabetes. Diabetes causes increased production of advanced glycation end products (AGEs), tissue adhesion inflammatory molecules, oxidative stress, pro-inflammatory cytokines. This inflammatory process may lead to a higher susceptibility to infections with poorer outcomes. Some studies did not find a clear correlation between diabetes and COVID-19 infection [8].

According to Abdi et al., who summarized the data on COVID-19 and diabetes, revealed 14.5% prevalence of diabetes in COVID patients. The prevalence was significantly higher in severe COVID-19 cases. According to International Diabetes Federation (IDF), the symptoms of COVID-19 infection were well developed in diabetes [9]. Also, they have oral manifestations that includes dysgeusia, erosion, ulcer, vesicle, bulla, pustule, depapillated, fissured tongue, pigmentation, white or red plaque, papule, macule, halitosis, hemorrhagic crust, petechiae, necrosis, swelling, erythematous area, aphthous stomatitis, candidiasis, herpetiform lesions, vasculitis, drug eruption, erythema multiforme-like, Kawasaki-like, mucositis, necrotizing periodontal disease, angular cheilitis, angina bullosa-like, Melkerson-Rosenthal syndrome, and atypical Sweet syndrome [3,10,11]. COVID-19 also causes secondary infection affecting the respiratory system, blood-stream, and urinary tract [12]. In our case, the patient had symptoms such as fever and breathlessness and uncontrolled diabetes, who during the management of COVID-19 infection had developed mucormycosis. Although it is not confirmed that COVID-19 has a direct correlation with the development of mucormycosis, a few cases have been reported with its manifestations. COVID-19 infection has been causing the immune system to be compromised by reducing T lymphocytes, altering the innate immunity and increasing the blood glucose levels, which serve as an opportunistic platform for the development of mucormycosis [4,12-14].

Mucormycosis is an opportunistic fungal infection and factors like uncontrolled diabetes and immunocompromised situations can increase the chances of infection. Mucormycosis is caused by the fungi of the order Mucorales of the subphylum Mucoromycotina (formerly known as the class Zygomycetes). Infection caused by the Mucorales is termed as mucormycosis [15]. Seven families of order Mucorales can cause mucormycosis [6]. In the Western hemisphere, Rhizopus oryzae (in the family Mucoraceae) is the most common cause. Other fungi include Rhizopus microsporus, Rhizomucor, Mucor and Actinomuco of Mucoraceae family, Lichtheimia of Lichtheimiaceae family, Cunninghamella of Cunninghamella ceae family, Cokeromyces of Thamnidiaceae family, Mortierella of Mortierellaceae family, Saksenaea, Apophysomyces of Saksenaceae family and Syncephalastrum of Syncephalastraceae family. Mucorales like Acrogenotheca elegans and Mucor irregularis is the major cause of disease in India and in China, respectively [4,6,16].

Humans are constantly exposed to Mucorales. Risk factors for increased chances of acquiring mucormycosis are diseases like leukaemia, lymphoma, multiple myeloma, neutropenia, diabetes, cirrhosis, and acute renal failure; therapy includes antineoplastic agents, corticosteroids, antibiotics, immunosuppressants, IV drug abuse, radiation, and deferoxamine, and transplantation of solid organ, bone marrow, peripheral blood stem cell and other conditions like burns, trauma, and malnutrition [5,15,17]. On analyzing 929 cases from the English literature from 1940 to 2003, Roden et al. reported that mucormycosis was developed due to trauma (34%), diabetes (16%), malnutrition (7%), burn (6%), solid organ transplant (3.41%), deferoxamine use (2.76%), neutropenia (2.28%), and injection drug use (0.15%) cases [18]. Mignogna et al. analyzed that 73% of cutaneous mucormycosis had various risk factors, including trauma, burn, and surgery [15].

Mucormycosis is acquired by immune suppression or acidosis, invasion of spores through inhalation, trauma, surgical cuts, catheters or infarction of artery or iron chelation, decreased phagocytic function. Deferoxamine causes iron chelation, which acts as a siderophore by increasing the uptake of iron by fungus, stimulating its growth, and leading to tissue invasion. When the organism invades the blood vessel, it initiates clotting, which causes infarction of the vessels. Infarction leads to tissue death, which results in the development of black necrotic tissue in the palate, nares, or orbit. Necrosis increases the pH, which aids in the growth of organisms. The development of fungemia and the disseminated form are rapid due to angioinvasion, which causes difficulty in antifungal therapy [5,19,20].

The association between COVID-19 and diabetes is either due to chronic inflammation, immune dysregulation, increased coagulation activity, and potential direct pancreatic damage by the virus. The mechanism that causes mucormycosis in hyperglycemic conditions is disruption of the innate immune mechanism produced by iron-binding proteins, tissue penetration and endothelial cell damage caused by increased expression of GRP78 in diabetic ketoacidosis (DKA), impairment of phagocytic function [6,20].

Mucormycosis based on anatomic site and clinical presentation can be divided into six categories: rhino-orbital-cerebral, pulmonary, cutaneous, gastrointestinal, disseminated, and miscellaneous. Rhino-orbital-cerebral form is most common in patients with DKA. Leukemic patients receiving chemotherapy and patients undergoing hematopoietic stem cell transplantation (HSCT) are more prone to the development of pulmonary mucormycosis [6].

Mucormycosis can present with a headache, fever, facial swelling, periorbital edema, pain, black eschar nasal discharge, proptosis, chemosis, sinusitis, and necrotic nasal concha. Intraorally, they can represent with ulceration that can be large and deep, leading to necrosis and pain. The most common site of involvement is palate followed by gingiva, lip, and alveolar ridge [21-23]. Definitive diagnosis of mucormycosis is based on the biopsy followed by histopathological examination as it is the most sensitive and specific modality. It reveals thick-walled, ribbon-like, aseptate hyphal elements that branch at right angles. Periodic acid-Schiff or Grocott’s silver methenamine stain highlights the mucorales and visualizes it more effectively. If the mucorales are high in number, they can be visualized using hematoxylin and eosin. Histopathologically, the differential diagnosis would be aspergillus and scedosporium, which shows branching at an acute angle and thinner septae. Polymerase chain reaction (PCR) can identify fungal DNA from the tissue samples and may improve the diagnostic ability [6,24].

Rhino-orbital mucormycosis under CT or magnetic resonance imaging (MRI) imaging shows features of sinusitis that cannot be differentiated from bacterial sinusitis [6]. MRI can show early vascular invasion, perineural and intracranial spread. CT is best at detecting bony erosions and necrosis [20]. Endoscopy or surgical exploration is suggested in the suspected area of infection. Treatment should be initiated even before confirming the diagnosis when suspecting mucormycosis [6].

Management of mucormycosis involves a combination of surgical debridement of the affected tissue and antifungal therapy along with elimination of risk factors. Lipid formulation of amphotericin B (Liposomal amphotericin B) and amphotericin B lipid complex (ABLC) is the drug of choice as initial therapy. Amphotericin B deoxycholate is the only licensed drug for the management, though it is highly nephrotoxic. Step down therapy is given when the patient responds to lipid formulation of amphotericin B whereas when the patient does not respond or who cannot tolerate the initial therapy, salvage therapy is given. Posaconazole or isavuconazole are the drugs used in the step down or in salvage therapy. Antifungal therapy is indicated till the patient is free of infection and immunosuppression is stabilized [6,15,20]. The combination of posaconazole and amphotericin is not yet supported by guidelines [16,20]. Patients often undergo extensive debridement surgery, which can lead to a disfigured face.

Hyperbaric oxygen (HBO) therapy given as an adjuvant therapy can increase oxygen saturation level and improve vascularity, promotes cytokines secretion, and stimulates the growth of new blood vessel. Mucormycosis causes angioinvasion leading to tissue necrosis and thrombosed blood vessels. HBO given post-surgically can aid in the formation of granulation tissue and bone healing. Other therapies include adjunctive cytokines and iron chelation [25]. The mortality rate of mucormycosis varies from 16% to 100% with cutaneous form having 17%, rhino-cerebral form having 67%, pulmonary form having 83%, and gastrointestinal form having 100% [24].

## Conclusions

COVID-19 infection is a novel infection affecting worldwide causing systemic and oral manifestations. Massive use of antibiotics, antibodies, steroids during the management of COVID-19 have caused increase in several bacterial, fungal, and viral infections. Increased blood glucose levels in COVID-19 infection in turn serves as a risk factor for development of mucormycosis. Physicians should be aware of secondary infections produced during COVID-19 infection and be able to identify as early as possible. Delay in the identification or delay in initiation of the management will lead to extensive progression of the disease as seen in our case. Hence, interdisciplinary action is needed for proper management. Oral physician/oral medicine specialty being the first line dental physician to screen, diagnose and plan the process of management, plays an important role.

## References

[1] Azarpazhooh MR, Morovatdar N, Avan A (2020). COVID-19 pandemic and burden of non-communicable diseases: an ecological study on data of 185 countries. J Stroke Cerebrovasc Dis.

[2] Shah VK, Firmal P, Alam A, Ganguly D, Chattopadhyay S (2020). Overview of immune response during SARS-CoV-2 infection: lessons from the past. Front Immunol.

[3] Santos JAD, Normando AGC, da Silva RLC, De Paula RM, Cembranel AC, Santos-Silva AR, Guerra ENS (2020). Oral mucosal lesions in a COVID-19 patient: new signs or secondary manifestations?. Int J Infect Dis.

[4] Sen M, Lahane S, Lahane TP, Parekh R, Honavar SG (2021). Mucor in a viral land: a tale of two pathogens. Indian J Ophthalmol.

[5] Arani R, Shareef SNHA, Khanam HMK (2019). Mucormycotic osteomyelitis involving the maxilla: a rare case report and review of the literature. Case Rep Infect Dis.

[6] Parasher A (2021). COVID-19: current understanding of its pathophysiology, clinical presentation and treatment. Postgrad Med J.

[7] Brandão TB, Gueiros LA, Melo TS (2021). Oral lesions in patients with SARS-CoV-2 infection: could the oral cavity be a target organ?. Oral Surg Oral Med Oral Pathol Oral Radiol.

[8] Hussain A, Bhowmik B, do Vale Moreira NC (2020). COVID-19 and diabetes: kowledge in progress. Diabetes Res Clin Pract.

[9] Abdi A, Jalilian M, Sarbarzeh PA, Vlaisavljevic Z (2020). Diabetes and COVID-19: a systematic review on the current evidences. Diabetes Res Clin Pract.

[10] Tapia ROC, Labrador AJP, Guimaraes DM, Valdez LHM (2020). Oral mucosal lesions in patients with SARS-CoV-2 infection. Report of four cases. Are they a true sign of COVID-19 disease?. Spec Care Dentist.

[11] Iranmanesh B, Khalili M, Amiri R, Zartab H, Aflatoonian M (2021). Oral manifestations of COVID-19 disease: a review article. Dermatol Ther.

[12] Zhang H, Zhang Y, Wu J (2020). Risks and features of secondary infections in severe and critical ill COVID-19 patients. Emerg Microbes Infect.

[13] Mehta S, Pandey A (2020). Rhino-orbital mucormycosis associated with COVID-19. Cureus.

[14] Pasero D, Sanna S, Liperi C (2020). A challenging complication following SARS-CoV-2 infection: a case of pulmonary mucormycosis [PREPRINT]. Infection.

[15] Mignogna MD, Fortuna G, Leuci S, Adamo D, Ruoppo E, Siano M, Mariani U (2011). Mucormycosis in immunocompetent patients: a case-series of patients with maxillary sinus involvement and a critical review of the literature. Int J Infect Dis.

[16] Hernández JL, Buckley CJ (2021). Mucormycosis.

[17] Skiada A, Pavleas I, Drogari-Apiranthitou M (2020). Epidemiology and diagnosis of mucormycosis: an update. J Fungi (Basel).

[18] Roden MM, Zaoutis TE, Buchanan WL (2005). Epidemiology and outcome of zygomycosis: a review of 929 reported cases. Clin Infect Dis.

[19] Quandahl R, Jan AH, Cooper JS (2021). Hyperbaric Zygomycotic Infections. http://www.ncbi.nlm.nih.gov/books/NBK493208/.

[20] Ak AK, Gupta V (2021). Rhino-orbital Cerebral Mucormycosis. http://www.ncbi.nlm.nih.gov/books/NBK557429/.

[21] Mohamed MS, Abdel-Motaleb HY, Mobarak FA (2015). Management of rhino-orbital mucormycosis. Saudi Med J.

[22] Doni BR, Peerapur BV, Thotappa LH, Hippargi SB (2011). Sequence of oral manifestations in rhino-maxillary mucormycosis. Indian J Dent Res.

[23] Burket LW, Greenberg MS, Glick M, Ship JA (2008). Burket’s Oral Medicine, 11th ed. 11th ed. Hamilton, Ont: BC Decker.

[24] Rai S, Misra D, Misra A, Jain A, Jain P, Dhawan A (2018). Palatal mucormycosis masquerading as bacterial and fungal osteomyelitis: a rare case report. Contemp Clin Dent.

[25] Tragiannidis A, Groll AH (2009). Hyperbaric oxygen therapy and other adjunctive treatments for zygomycosis. Clin Microbiol Infect.

